# Associations Between Nurse–Patient Interaction and Sense of Coherence Among Cognitively Intact Nursing Home Residents

**DOI:** 10.1177/0898010120942965

**Published:** 2020-07-23

**Authors:** Jorunn Drageset, Siw Eriksen Taasen, Birgitte Espehaug, Britt Moene Kuven, Wenche Mjanger Eide, Beate André, Eva Rinnan, Gørill Haugan

**Affiliations:** Western Norway University of Applied Sciences; University of Bergen; Western Norway University of Applied Sciences; Norwegian University of Science and Technology; Norwegian University of Science and Technology; Nord University

**Keywords:** nurse–patient interaction, holistic nursing, nursing home, older, sense of coherence

## Abstract

**Aim:** To investigate the association between nurse–patient interaction and sense of coherence among cognitively intact nursing home residents. **Method:** In a cross-sectional design, data were collected in 2017 and 2018 using the Nurse–Patient Interaction Scale (NPIS) and the 13-item Sense of Coherence Scale (SOC-13). Of the 204 cognitively intact nursing home residents who met the inclusion criteria, 188 (92%) participated, representing 27 nursing homes. Multiple regression in a general linear model estimated the possible effects of the 14 NPIS items on SOC-13 sum score, the possible effects of the NPIS (sum score) on SOC-13 (sum score) as well as on the subdimensions of SOC-13, comprehensibility, meaningfulness, and manageability (both without and with adjusting for sex and age). **Results:** Four of the 14 NPIS items revealed highly significant correlations with SOC-13 (sum score; unadjusted and adjusted for age and gender). Furthermore, the analysis adjusted for age and gender showed significant associations for NPIS (sum score) with SOC-13 (sum score), manageability, and comprehensibility. The correlation between NPIS and meaningfulness was not statistically significant. **Conclusion:** Nurse–patient interaction is significantly associated with SOC-13 and its subdimensions of comprehensibility and manageability but not meaningfulness. Nurse–patient interaction might be an important resource in relation to residents’ sense of coherence and its subdimensions.

## Introduction

The number of people 60 years or older in the world is increasing (World Health Organization, 2018), rising from 900 million (12% of the world population) in 2015 to 2 billion (22%) in 2050. The proportion of people 80 years and older will increase more than that of people 60 to 79 years old. Today, 125 million people are 80 years or older (World Health Organization, 2018). Increased age leads to functional decline and chronic comorbidity (Fabbri et al., 2015), resulting in increased need for health care services and long-term care.

In Norway, about 7.2% of the people older than 67 years and 9.1% of those older than 80 years live in facilities offering a high level of residential care, described as nursing homes, and most of the residents live there in the last phase of their life. Moving to a nursing home may be related to numerous losses, such as losing one’s home, friends, and family. This makes people realize that their life is ending, and the dependence on other people is a reality that must be accepted in a culture in which autonomy is rated highly (Chow et al., 2004).

The characteristics of the nursing home population are chronic illnesses, multiple diagnoses, a persistent symptom burden (Drageset et al., 2009; Haugan, 2014a), dependence on help to carry out the activities of daily living (Drageset et al., 2011), and 80% with dementia (Bergh et al., 2012). These life challenges combined with most residents being cognitively impaired (Bergh et al., 2012) may influence the individual’s inner strength and ability to adapt and cope (Nygren et al., 2005). Hence, a holistic approach to nursing home care is highly needed, requiring attention to the nursing home residents as an entity of body–mind–spirit. In a holistic approach, sense of coherence might be a vital resource for the well-being among cognitively intact nursing home residents.

Sense of coherence is a central concept of Antonovsky’s holistic understanding of health as a steadily ongoing process or movement along a continuum between poor and good health and has been shown to influence coping (Lovheim et al., 2013). Antonovsky (1979, 1987) understood sense of coherence to represent a person’s confidence in having the resources needed to cope with challenges. Sense of coherence signifies a global orientation in life, involving the degree to which one has a pervasive and enduring but dynamic feeling of confidence. That is, the stimuli arising from one’s internal and external environments are structured, predictable, and explicable (comprehensibility); that one has the resources available to meet the demands posed by these stimuli (manageability); and that these demands are challenges worthy to engage in (meaningfulness; Antonovsky, 1979, 1987). Together, these components reflect the interactions of an individual with resources in the environment.

Among individuals with a low sense of coherence, major later life crises can weaken their sense of coherence, whereas a strong sense of coherence remains either stable or is only temporarily reduced (Antonovsky, 1979, 1987). According to Antonovsky (1987), general resistance resources such as material resources, knowledge and intelligence, ego identity, and social support are vital for a person’s sense of coherence. General resistance resources are shaped by life experiences and developed over one’s life span and acting as a resource for meaningful and coherent life experiences that reinforce a sense of coherence (Antonovsky, 1987). This means that a person’s general resistance resources support the individual in making sense of the various stressors in life, thus strengthening the sense of coherence. When confronted with a stressor, people with a strong sense of coherence can use their general resistance resources to manage the situation successfully. A low sense of coherence indicates greater vulnerability (Antonovsky 1979, 1987). In a holistic perspective, it is therefore vital to assess residents’ sense of coherence in long-term nursing home care. This means to support individuals’ well-being through a broader perspective that includes unity of body, mind, and emotion, adjusted to the environment and how these interact and affect each individual’s ability to grow and heal for better health, quality of life, and a perception of wellness (Guzzetta, 2005).

Nursing home residents may experience life challenges; the relocation to a nursing home might represent various stressors such as loss of autonomy and home. In these cases, health care personnel can be defined as part of the general resistance resources and thus function as a vital health-promoting resource. Kvale and Synnes (2013) found that, by means of good care, health care personnel performed to be a vital resource, which strengthened cancer patients’ general resistance resources in a stressful life situation. Nurses, doctors, family, and friends functioned as vital resources at their disposal when needed (Kvale & Synnes, 2013). Likewise, Lung and Liu (2016) found that daily positive interactions between nursing assistants and nursing home residents improved the psychosocial well-being of the residents and encouraged them to cooperate during the delivery of care, which improved their overall health. Another study among nurses (Lillekroken et al., 2015) investigated salutary factors that encouraged sense of coherence among people with dementia. The main salutary dimensions were *establishing continuity, ensuring predictability*, and *building confidence*. A systematic review among people 65 years and older (Tan et al., 2014) found that the use of general resistance resources such as appraisal, coping strategies, and social support were correlated with their sense of coherence, perceived holistic health, and quality of life.

Many consider the nurse–patient relationship to be the core of nursing (Halldorsdottir, 2008; Warelow et al., 2008). A nurse who is competent and who connects with the patient in an empowering way influences the patient positively and facilitates a sense of well-being and health, hope, meaning-in-life, and satisfaction (Calong & Soriano, 2018; Dwyer et al., 2008; Halldorsdottir, 2008; Haugan, 2014b). In nursing home care, caregivers and residents should be able to communicate their goals in an open atmosphere characterized by empathy and respect, acknowledging and supporting the residents’ right to actively participate (Swenne & Skytt, 2014). The process of caring is also described as an interactive and intersubjective human process that occurs during moments of shared vulnerability between two or more people (Calong & Soriano, 2018). Most nursing home residents have few relationships, which might represent limited possibilities for dialogue, self-reflection, and connectedness. To meet the residents’ specific needs holistically, the nurse–patient interaction is essential (Cahill et al., 2012; Haugan et al., 2016). Holistic care includes caring for each patient as a whole human being, including emotional, physical, social, and spiritual/existential dimension, as well as being present together with that patient (Quinn, 2005).

Recent studies among nursing home residents have shown a signiﬁcant impact of the nurse–patient interaction in relation to intrapersonal and interpersonal transcendence (Haugan et al., 2012; Haugan, Hanssen, & Moksnes, 2013), hope (Haugan, Moksnes, & Espnes, 2013), perceived meaning-in-life (Haugan, 2014a), anxiety and depression (Haugan, Innstrand, & Moksnes, 2013), quality of life (Haugan et al., 2016), and joy-of-life (Haugan et al., 2019). Positive relationships, quality of care, and nurse–patient interaction have been found to act as core aspects contributing to thriving and well-being among long-term nursing home residents (Aiken et al., 2012; Bergland & Kirkevold, 2005; Edvardsson et al., 2017). Nurses represent nursing home residents’ daily opportunities for connectedness and communication. Hence, the nurse–patient interaction might therefore be essential to the residents’ sense of coherence. Accordingly, the nurse–patient interaction may perform as a general resistance resource and thereby strengthen the residents’ sense of coherence.

To summarize, studies have shown a positive association between nurse–patient interaction and hope, self-transcendence, meaning-in-life, quality of life, and joy-of-life, and a negative association with anxiety and depression (Haugan, 2014c; Haugan, Hanssen, & Moksnes, 2013, Haugan et al., 2016, Haugan et al., 2019), whereas others have focused on residents’ satisfaction with care (Calong & Soriano, 2018). Moreover, research has shown that health care personnel by means of good care can function as a vital resource for well-being among cancer patients (Kvale & Synnes, 2013; Lillekroken et al., 2015). Nevertheless, the evidence on how nurse–patient interaction is related to sense of coherence in the nursing home population is sparse. In addition, even though some evidence is emerging supporting a positive impact of nurse–patient interaction on nursing home residents’ hope, self-transcendence, meaning-in-life, quality of life, and joy-of-life, as well as anxiety and depression, further studies are recommended. No previous study has assessed the relationship between nurse–patient interaction and sense of coherence among older cognitively intact nursing home residents.

## Aims

Therefore, the aim of this study was to contribute to this knowledge gap by investigating the association between nurse–patient interaction and sense of coherence.

The following hypotheses were tested:

**Hypothesis 1:** Sense of coherence (sum score of 13-item Sense of Coherence Scale [SOC-13]) is positively associated with each of the 14 Nurse–Patient Interaction Scale (NPIS) items measuring nurse–patient interaction.
**Hypothesis 2:** Sense of coherence (sum score of SOC-13) is positively associated with nurse–patient interaction (sum score of NPIS).
**Hypothesis 3:** The three dimensions of sense of coherence (comprehensibility, manageability, and meaningfulness) in SOC-13 are positively associated with nurse–patient interaction (sum score of NPIS).

## Method

### The Study Setting

In Norway, most nursing homes are owned and operated by the public sector as part of the municipal health services; hence, the municipalities fund nursing homes. Privately owned nursing homes are scarce. In addition, Norway’s health care system, including nursing homes, are rather uniform and standardized, with only slight structural differences. Local boards determine all admissions to private or public nursing homes. Regardless of ownership status, the municipality is the only source of money, which involves a flat rate per bed. Beyond this, the nursing homes are highly autonomous regarding how they organize their nursing and health care, as long as legal requirements and norms are met. Staffing ratios are quite similar across the municipalities. Norway’s nursing home population comprises individuals with multiple diagnoses who depend on various types of assistance with the basic activities of daily living; consequently, most people who enter a nursing home need extensive assistance and have a complex clinical picture. In this study, the participants represented 27 nursing homes located in two large urban municipalities: (1) Central Norway (*n* = 88) and (2) Western Norway (*n* = 100).

### Design, Data Collection, and Procedure

This study used a cross-sectional design. The data were collected between 2017 and 2018 from 27 nursing homes in two large urban municipalities in Norway (in Central and Western Norway) that met the inclusion criteria: (1) the municipalities’ (local boards’) decision on eligibility for long-term nursing home care, (2) residing for 3 months or longer, (3) competence to give informed consent recognized by the responsible doctor and nurse, and (4) capable of being interviewed recognized by the responsible doctor and nurse. The exclusion criteria were as follows: (1) short-term care, (2) rehabilitation stays, and (3) diagnosed with dementia; a responsible nurse at the ward identified from the medical records if the patients were diagnosed with dementia. Of the 204 cognitively intact nursing home residents who met the inclusion criteria, 188 (92%) participated.

The nurse at the ward presented the potential participants with oral and written information about the study, their rights as participants, and their right to withdraw at any time.

Six researchers (three in each part of Norway) with identical professional background (registered nurse, MSc trained and experienced in communication with older people, and teaching gerontology at an advanced level) were trained to conduct the face-to-face interviews as identically as possible. The researchers were required to ask the questions on the instruments since many residents could not read or write clearly.

The Regional Committee for Medical and Health Research Ethics in Central Norway (Ref. No. 2014/2000/REK Central) approved the study, as did the management units at the 27 nursing homes. Each participant provided written informed consent.

### Measures

The questionnaires were part of a battery of seven scales comprising 120 items.

#### Demographic Variables

Demographic data: Age, gender, and marital status were collected.

#### The Orientation to Life Questionnaire-13 Assessed Sense of Coherence

Based on the salutogenic health theory, Antonovsky (1987) developed the original 29-item Sense of Coherence Scale (SOC). Later, the 13-item version of the SOC was developed (SOC-13); we used the Norwegian version of the SOC-13, recently validated among cognitively intact nursing home residents (Drageset & Haugan, 2016). The 13 items are rated on a 7-point scale providing two anchoring verbal responses: *very seldom or never* and *very often*. The total score ranges from 13 to 91; higher scores indicate a stronger sense of coherence (Antonovsky, 1979, 1987). A systematic review of the validity and reliability of the SOC-13 (Eriksson & Lindström, 2005) showed that it is generally acceptable among older people.

#### The Nurse–Patient Interaction Scale Assessed Nursing Home Residents’ Perceived Nurse–Patient Interaction

The NPIS was developed in Norway to identify important characteristics of nursing home residents’ experiences of the nurse–patient interaction and validated in a nursing home population (Haugan, Hanssen, & Moksnes, 2013). The NPIS is a 10-point-scale ranging from 1 (*not at all*) to 10 (*very much*); higher numbers indicate better perceived nurse–patient interaction. Examples of NPIS items include having trust and confidence in the staff nurses, the experience of being taken seriously, experiences of being respected and recognized as a person, and being listened to and feeling good as a result of the nurse–patient interaction. The NPIS comprises 14 items identifying essential relational qualities stressed in the nursing literature (Haugan, Hanssen, & Moksnes, 2013) developed to measure the nursing home residents’ sense of well-being derived from the nurse–patient interaction (Finch, 2005; Hollinger-Samson & Pearson, 2000; Rchaidia et al., 2009). The NPIS has shown good psychometric properties with good content validity and reliability (Cronbach’s α = .92 among nursing home residents; Haugan, 2014c; Haugan et al., 2016) and a total mean score of 8.13 (*SD* = 1.6; Haugan et al., 2012).

### Data Analysis

Descriptive statistics for the demographic variables and the NPIS were calculated, and reliability of the latent constructs of NPIS and SOC-13 was assessed by using Cronbach’s alpha reliability coefficient.

Multiple general linear regression models assessed possible effects of each of the 14 NPIS-items on SOC-13 (sum score), of the NPIS (sum score) on SOC-13 (sum score), and the NPIS (sum score) on SOC subdimensions comprehensibility, meaningfulness, and manageability (both without and with adjusting for sex and age). We coded sex as a categorical variable and NPIS and age as continuous covariates (Table 2). We used SPSS for Windows (Version 18.0, 2014; Morgan & Griego, 1998) for all statistical analysis, applying a significance level of .05 throughout.

## Results

### Characteristics of the Participants

The participants’ ages ranged from 63 to 104 years, with a mean age of 87.4 years (*SD* = 8.57). The sample included 138 women (73%) and 50 men (27%), with a mean age of 88.3 years for the women (*SD* = 1.80) and 86.0 years (*SD* = 1.16) for the men. In total, 23 were married, 22 were cohabiting, 1 was single, 106 were widowed, and 36 were divorced. The mean length of stay in the nursing home when interviewed was 2.6 years for both sexes (range 0.3 to 10 years, table not shown). The mean SOC-13 score range for the total population was 56.7 to 67.9 (*SD* = 6.3). Cronbach’s alpha was .70.

### Associations Between Sense of Coherence Scale-13 Questionnaire Sum Score and Nurse–Patient Interaction Scale

In the unadjusted analysis, the items NPIS2 (“The nurses take me seriously,” *b* = 0.69, *p* = .002), NPIS4 (“The nurses understand me,” *b* = 0.60, *p* ≤ .001), NPIS5 (“The nurses make all possible efforts to relieve my ailments,” *b* = 0.67, *p* = .001), and NPIS9 (“The nurses are listening interestingly to me,” *b* = 0.44, *p* = .004) were statistically significantly correlated with the SOC-13 sum score (Table 1). The significant associations remain in the adjusted (for age and gender) analysis for NPIS2 (“The nurses take me seriously,” *b* = 0.58, *p* = .011), NPIS 4 (“The nurses understand me,” *b* = 0.60, *p* ≤ .001), NPIS5 (“The nurses make all possible effort to relieve my ailments,” *b* = 0.52, *p* = .013), and NPIS9 (“The nurses are listening interestingly to me,” *b* = 0.42, *p* = .008; Table 1). Cronbach’s alpha was .90.

**Table 1.** Univariate Analyzes, Unadjusted and Adjusted for Sex and Age for Nurse–Patient Interaction Scale (NPIS) Among 188 Cognitive Intact Nursing Home Patients

NPIS Item	Unadjusted			Adjusted		
	*M, SD*	*b*	*p* Value	*b*	95% CI	*p* Value
NPIS1: Having confidence and trust in the nurses	8.52, 2.13	0.39	.086	0.61	[−0.18, 0.74]	.234
NPIS2: The nurses take me seriously	8.44, 2.17	0.69	.002	0.58	[0.13, 1.02]	.011
NPIS3: Interaction with the nurses makes me feel good	8.03, 2.36	0.38	.058	0.29	[−0.12, 0.69]	.161
NPIS4: The nurses understand me	7.08, 2.70	0.60	.001	0.60	[0.25, 0.95]	.001
NPIS5: The nurses make all possible efforts to relieve my plagues	8.41, 2.31	0.67	.001	0.52	[0.11, 0.94]	.013
NPIS6: The nurses involve me in decisions regarding my daily life	6.99, 3.06	0.19	.227	0.14	[−0.17, 0.45]	.364
NPIS7: The nurses treat me with respect	8.54, 2.05	0.39	.100	0.29	[−0.16, 0.76]	.204
NPIS8: The nurses ask me how I am	6.96, 3.16	0.28	.059	0.26	[−0.04, 0.55]	.090
NPIS9: The nurses are listening interestingly to me	6.99, 3.06	0.44	.004	0.42	[0.11, 0.73]	.008
NPIS10: I often get hurt or sad from how the nurses interact	3.17, 2.84	−0.28	.102	−0.21	[−0.54, 0.12]	.213
NPIS11: Interaction with the nurses contributes to meaning in my life	7.59, 2.57	0.11	.566	0.03	[−0.34, 0.39]	.888
NPIS12: The nurses pay attention to me as a person	7.38, 2.85	0.29	.098	0.29	[−0.06, 0.65]	.101
NPIS13: I am satisﬁed with the communication with the nurses	8.15, 2.40	0.27	.180	0.16	[−0.24, 0.55]	.440
NPIS14: Interaction with nurses is the most important to my thriving	8.68, 2.03	0.10	.664	0.14	[−0.33, 0.61]	.557

*Note. b* = regression coefficient; CI = confidence interval.

### Associations Between SOC-13 Sum Score and NPIS Sum Score

In the unadjusted analysis, age was significantly associated with SOC-13 sum-score (*b* = 0.15, *p* = .01), whereas SOC-13 was significantly correlated with NPIS (*b* = 0.05, *p* = .01). The adjusted analysis revealed statistically significant associations between age and SOC-13 sum score (*b* = 0.15, *p* = .01) and between NPIS and SOC-13 sum score (*b* = 0.05, *p* = .04).

### Associations Between SOC-13 Subdimensions and NPIS Sum Score

#### Unadjusted Analysis

The unadjusted analysis disclosed significant associations between age and two of the SOC-13 dimensions: comprehensibility (*b* = 0.10, *p* = .03) and meaningfulness (*b* = 0.10, *p* ≤ .001). The associations between NPIS sum score and the three dimensions of SOC-13 revealed significant estimates: comprehensibility (*b* = 0.07, *p* ≤ .001) and manageability (*b* = −0.03, *p* = .03). The meaningfulness dimensions of the SOC-13 and NPIS sum scores were not significantly correlated (*b* = 0.01, *p* = .91; Table 2).

**Table 2.** Univariate Analyses for SOC-13 Sum Score and SOC-13 Subdimensions Among 188 Cognitive Intact Nursing Home Residents Unadjusted and Adjusted for Sex, Age, and NPIS.

Variable	Unadjusted				Adjusted			
	SOC-13 sum score, *b* [95% CI]	Comprehensibility, *b* [95% CI]	Manageability, *b* [95% CI]	Meaningfullness, *b* [95% CI]	SOC-13 sum score, *b* [95% CI]	Comprehensibility, *b* [95% CI]	Manageability, *b* [95% CI]	Meaningfullness, *b* [95% CI]
Sex								
Female	−0.15 [−2.33, 2.02]	−1.40 [−3.08, 0.29]	0.40 [−0.50, 1.30]	0.85 [0.34, 2.04]	−0.38 [−2.55, 1.79]	−1.48 [−3.03, 0.22]	0.40 [−0.50, 1.29]	0.85 [−0.35, 2.04]
Male	Reference	Reference	Reference	Reference	Reference	Reference	Reference	Reference
Age	0.15 [0.05, 0.26]	0.10 [0.01, 0.18]	−0.01 [−0.05, 0.04]	0.10 [0.04, 0.16]	0.15 [0.04, 0.27]	0.94 [0.01, 0.18]	0.48 [−0.05, 0.05]	0.10 [0.04, 0.16]
NPIS	0.05 [0.11, 0.09]	0.07 [0.04, 0.09]	−0.03 [−0.04, −0.01]	0.01 [−0.02, 0.02]	0.05 [0.01, 0.09]	0.07 [0.04, 0.09]	0.03 [−0.04, −0.01]	−0.00 [−0.02, 0.02]

*Note.* Age range = 63 to 104 years. SOC-13 = 13-item Sense of Coherence Scale; *b* = regression coefficient; CI = confidence interval; NPIS = Nurse–Patient Interaction Scale.

#### Adjusted Analysis

In adjusted analysis, the significant correlations between the SOC-13 dimensions manageability and comprehensibility and NPIS sum score remain in age (*b* = 0.48, *p* = .04 vs. *b* = 0.94, *p* = .003) and between the NPIS sum score and SOC-13 dimensions manageability and comprehensibility (*b* = 0.03, *p* = .002 vs. *b* = −0.07, *p* ≤ .001). The meaningfulness dimension of SOC-13 and NPIS sum score were not significantly correlated (*b* = −0.00, *p* = .90; Table 2).

## Discussion

We investigated the associations between SOC-13 and each of the NPIS 14 items, SOC-13, and the NPIS sum score, and the associations between NPIS sum score and the three dimensions of SOC-13 (comprehensibility, manageability, and meaningfulness) among the cognitively intact nursing home residents.

In the adjusted analysis, the study suggested a significant association between SOC-13 (sum score) and NPIS. Moreover, the most vital NPIS items in relation to SOC-13 were NPIS2 (“The nurses take me seriously”), NPIS4 (“The nurses understand me”), NPIS5 (“The nurses make all possible effort to relieve my ailments”), and NPIS9 (“The nurses are listening interestingly to me”). Furthermore, our results showed significant correlations between age and the SOC-13 dimensions of manageability and comprehensibility and between NPIS and the SOC-13 dimensions manageability and comprehensibility. NPIS was not significantly associated with the meaningful dimension of SOC-13.

The findings imply that nurse–patient interaction is a vital resource in strengthening nursing home residents’ sense of coherence, with the aspects “The nurses take me seriously,” “The nurses understand me,” “The nurses make all possible effort to relieve my ailments” being essential, and “The nurses are listening interestingly to me” being important. Listening to the residents, supporting them, and taking them seriously seem to strengthen the sense of coherence. The nurse–patient relationship is highlighted as the core of nursing and for empowering patients in a positive life-giving way (Halldorsdottir, 2008). A nurse–patient relationship that influences the patients positively gives them a sense of well-being and health (Halldorsdottir, 2008). In this relationship, the patients should have the possibility to communicate their hope and meaning-in-life in an open and empathic atmosphere (Swenne & Skytt, 2014), which is essential for meeting the residents’ specific needs and improving their well-being.

Consequently, the nurse–patient relationship may function as a vital resource for the well-being among nursing home residents (Haugan et al., 2016; Kvale & Synnes, 2013). In this context, the nurse–patient relationship may be considered as an aspect of the residents’ general resistance resources as described by Antonovsky (1987). Thus, general resistance resources may support an individual’s sense of coherence. Furthermore, an individual’s sense of coherence may contribute to mobilizing general resistance resources in order to enhance tension management. Volanen et al. (2004) found that older adults’ ability to receive social support and their satisfaction with this support contributed to their level of sense of coherence. However, because of the cross-sectional design, our result may possibly be of reciprocal relationships between NPIS and SOC-13, suggesting that a high level of nurse–patient interaction may contribute to a strong sense of coherence, and higher sense of coherence may increase the NPIS score.

Our results show that nurse–patient interaction was associated with comprehensibility. This finding suggests that nurses communicate in a way that is easy for the residents to understand, which, in turn, makes the conditions in nursing homes more comprehensible and predictable. Communication and information, which are understandable and ensure continuity and predictability regarding caring, are essential for strengthening nursing home residents’ sense of coherence and are thus described as salutary factors (Lillekroken et al., 2015). Furthermore, regarding the significant association between NPIS and manageability, our results indicate that nurses identify residents’ previous strengths and the internal and external resources they currently have available and help them to use these resources despite any limitations. Nursing home residents might use internal resources in their daily routines such as eating and going to bed and then experience self-determination and control of their daily life (Andersson et al., 2007). In addition, social support from families may act as a resource by providing the nursing home health professionals with central information about the residents’ interests, hobbies, and so on and thereby serve as both direct and indirect resources for the well-being and health of the residents. Consequently, social support acts as an important general resistance resource. Tan et al. (2014) found that social support, viewed as part of nursing home residents’ general resistance resources, was correlated with residents’ sense of coherence and their multidimensional well-being (physical, emotional, social, and spiritual/existential well-being). Furthermore, family members have an important role in the connection between nursing staff and residents by promoting integration of the family, improving information, increasing trust, and contributing to mutual understanding of expectations and goals that ultimately improve the care for residents with cognitive impairment (Utley-Smith et al., 2009).

The knowledge that social support is available is often enough to be a coping resource and to increase a person’s inner strength. The quality of social support has significant and close emotional ties that Antonovsky (1979, 1987) defines as being especially important. People who have good close ties with other people resolve tension more easily than those who lack that quality in their relationships.

Moreover, this study found a statistically significant correlation between nurse–patient interaction and SOC-13 meaningfulness in the unadjusted analysis but not in the adjusted analyses. The meaningfulness dimension of SOC-13 involves being motivated and having a desire to cope with internal and external stimuli, which are perceived to be challenges that are worthy to engage in (Antonovsky, 1979, 1987). Our findings contrast with earlier studies among nursing home residents. Haugan (2014b, 2014c) found significant associations between NPIS and meaning-in-life (measured by the Purpose-in-Life test) among older people in nursing homes. One explanation may be the fact that different measures are used for meaning-in-life. Despite this, earlier studies have highlighted that nursing home residents perceived nurse–patient interaction as being essential for meaning-in-life and multidimensional well-being (Haugan, 2014c; Haugan et al., 2016), and physical and mental well-being, belonging, personally treasured activities, and spiritual closeness have been shown to promote meaning and purpose in life (Drageset et al., 2017, Rinnan et al., 2018).

Consequently, maintaining nursing home residents’ interests and values seems to be a useful strategy for supporting meaningfulness. Antonovsky (1987) suggested four areas in which people need to invest to maintain a sense of meaningfulness: feelings, interpersonal relationships, employment, and existential values. Relationships providing emotional support are shown to be essential for loneliness (Drageset et al., 2010; Drageset et al., 2015; Victor & Bowling, 2012) and physical and mental well-being (Bergland & Kirkevold, 2008; Drageset et al., 2017) among nursing home residents. Likewise, relationships with caregivers are important for residents’ well-being (Bergland & Kirkevold, 2005), and talking is an important activity for nursing home residents’ life satisfaction (Andersson et al., 2007). Accordingly, in accordance with the goal of holistic nursing care (Guzzetta, 2005), nurse–patient interaction may represent a resource for hope, meaning-in-life, satisfaction, and multidimensional well-being in this population (Calong & Soriano, 2018; Dwyer et al., 2008; Haugan, 2014b).

## Limitations and Strength

The association between nurse–patient interaction and sense of coherence has not been investigated among nursing home residents previously. Thus, a strength of this study is the contribution of new knowledge about nurse–patient interaction and sense of coherence, both of which are vital to nursing home residents’ well-being. Furthermore, the high response rate (92%) and the use of well-validated instruments strengthen these results. The reliability of the measurements used was good, showing good estimates for Cronbach’s α .70 (SOC-13) and .90 (NPIS). These estimates are comparable with other studies using SOC-13 and NPIS among nursing home residents. The NPIS mean score in this study is in accordance with a previous Norwegian nursing home study reporting a total mean score of 8.13 (*SD* = 1.6; Haugan et al., 2012).

However, there are limitations. Concerning the data collection, responding to 113 items might be tiring for nursing home residents resulting in fatigue during the interviews representing a response bias. To avoid tiring the participants, the interview had planned breaks, and if necessary, the interview was divided and continued at another time. To counteract monotony and possible stereotypical answers, the question order and length of the interview were performed to reduce the respondent burden. In addition, all interviewers were trained to ensure the reliability and validity of the data obtained through the face-to-face interviews. Furthermore, the study was based on cross-sectional data, which do not enable conclusions about causality. In addition, the Norwegian health care system is rather homogeneous (uniform) and standardized, with only slight structural differences; the nursing homes in Western and Mid-Norway are therefore likely representative of cognitively intact nursing home residents in Norway, in general. The present study sample included cognitively intact nursing home residents. Therefore, the present results cannot be generalized to the nursing home population in general, which also includes a great number of individuals with dementia.

## Conclusions and Future Directions

Nurse–patient interaction was significantly related to sense of coherence and two of its subdimensions, comprehensibility and manageability, but not meaningfulness. Nurse–patient interaction might be an important resource in relation to residents’ sense of coherence. Further research should continue to explore how nurse–patient interaction can promote sense of coherence in nursing home residents, as a guide for holistic nursing interventions. Research can contribute to such knowledge about holistic nursing care, both theoretically and clinically. Moreover, both nurse–patient interaction and sense of coherence are central dimensions correlated to well-being, which is the primary goal in holistic nursing care.

## Implications for Holistic Nursing

Holistic nursing care should develop nursing strategies to promote residents’ sense of coherence. In this situation, nurses should recognize that the nurse–patient interaction is associated with sense of coherence and its subdimensions, comprehensibility and manageability, and pay attention to nurse–patient interaction as an important, integral part of the holistic practices in promoting sense of coherence. In relation to comprehensibility, residents could be informed about and understand the nature of their care to meet residents’ needs. In relation to manageability, nurses need to make the residents aware of the resources available and help use them and be aware of residents’ need for and desire to feel a sense of control over their daily lives such as going to bed, eating, and caring routines. A sense of control may contribute to the experience of manageability.

To facilitate residents’ meaningfulness, nurses should be aware of the importance in supporting and facilitating meaningful activities in the nursing home, especially activities the older adult previously valued but had to give up after moving to the nursing home. These could be occupational therapy and participating in the political, cultural, and religious arenas. Furthermore, professionals could support residents in maintaining their close relationships and by providing emotional support (meaningfulness). Nurses should be provided opportunities for improving their skills and competence in communicating and interacting. The relational qualities embedded in the nurse–patient interaction such as listening, empathetic understanding, respecting, accepting, and acknowledging the resident should be essential aspects of nursing home care and emphasized in clinical practice. Special attention should be paid to listening, taking the resident seriously, understanding, and relieving infirmity. The behavior of the nursing home staff as part of the interaction with the residents will affect, positively or negatively, nursing home residents’ sense of coherence and, thus, well-being.

In strengthening nursing home residents’ sense of coherence and thereby well-being, nurse–patient interaction should get more attention both in research and education as a vital integral part of the nursing process.
